# Topoisomerases and cancer chemotherapy: recent advances and unanswered questions

**DOI:** 10.12688/f1000research.20201.1

**Published:** 2019-09-30

**Authors:** Mary-Ann Bjornsti, Scott H. Kaufmann

**Affiliations:** 1 Department of Pharmacology and Toxicology, University of Alabama at Birmingham, Birmingham, AL, 35294-0019, USA; 2 Departments of Oncology and Molecular Pharmacolgy & Experimental Therapeutics, Mayo Clinic, Rochester, MN, 55905, USA

**Keywords:** DNA supercoiling, DNA-protein crosslink, DNA-activated protease, topoisomerase poison, chromatin organization

## Abstract

DNA topoisomerases are enzymes that catalyze changes in the torsional and flexural strain of DNA molecules. Earlier studies implicated these enzymes in a variety of processes in both prokaryotes and eukaryotes, including DNA replication, transcription, recombination, and chromosome segregation. Studies performed over the past 3 years have provided new insight into the roles of various topoisomerases in maintaining eukaryotic chromosome structure and facilitating the decatenation of daughter chromosomes at cell division. In addition, recent studies have demonstrated that the incorporation of ribonucleotides into DNA results in trapping of topoisomerase I (TOP1)–DNA covalent complexes during aborted ribonucleotide removal. Importantly, such trapped TOP1–DNA covalent complexes, formed either during ribonucleotide removal or as a consequence of drug action, activate several repair processes, including processes involving the recently described nuclear proteases SPARTAN and GCNA-1. A variety of new TOP1 inhibitors and formulations, including antibody–drug conjugates and PEGylated complexes, exert their anticancer effects by also trapping these TOP1–DNA covalent complexes. Here we review recent developments and identify further questions raised by these new findings.

## Introduction

The helical structure of duplex DNA provides a physical basis for the faithful duplication and deciphering of genetic information while also ensuring DNA strand integrity. The intertwining of the two complementary polynucleotide strands is stabilized by hydrogen bonding and stacking interactions between the hydrophobic bases. Yet these features also impose topological constraints during processes involving DNA ^1– 4^. For example, during DNA replication, each strand serves as a template for polymerization of a complementary strand. However, the progressive unwinding of antiparallel DNA strands may cause overwinding (positive supercoiling) ahead of the replication fork and intertwining of daughter DNA molecules (precatenanes) behind the fork. Similar topological considerations apply to transcription, which induces local unwinding (negative supercoiling) of the DNA helix behind the advancing RNA polymerase complex and positive supercoiling ahead of it.

The ability of cells to resolve local domains of DNA supercoiling and separate multiply intertwined DNA molecules is critical for gene expression, recombination, DNA replication, and chromosome segregation, yet it must be achieved while still maintaining chromosomal integrity. Solutions to these problems involve a family of enzymes called DNA topoisomerases, which catalyze changes in the linkage of DNA strands (or helices) by nicking one or both strands of the DNA duplex and, at the same time, becoming covalently linked to one end of the cleaved DNA through a phosphotyrosyl bond. After another DNA strand (or duplex) is passed through the protein-linked break(s) to produce a change in DNA topology, the original phosphodiester bond is religated to restore integrity of the DNA backbone.

**Figure 1.**
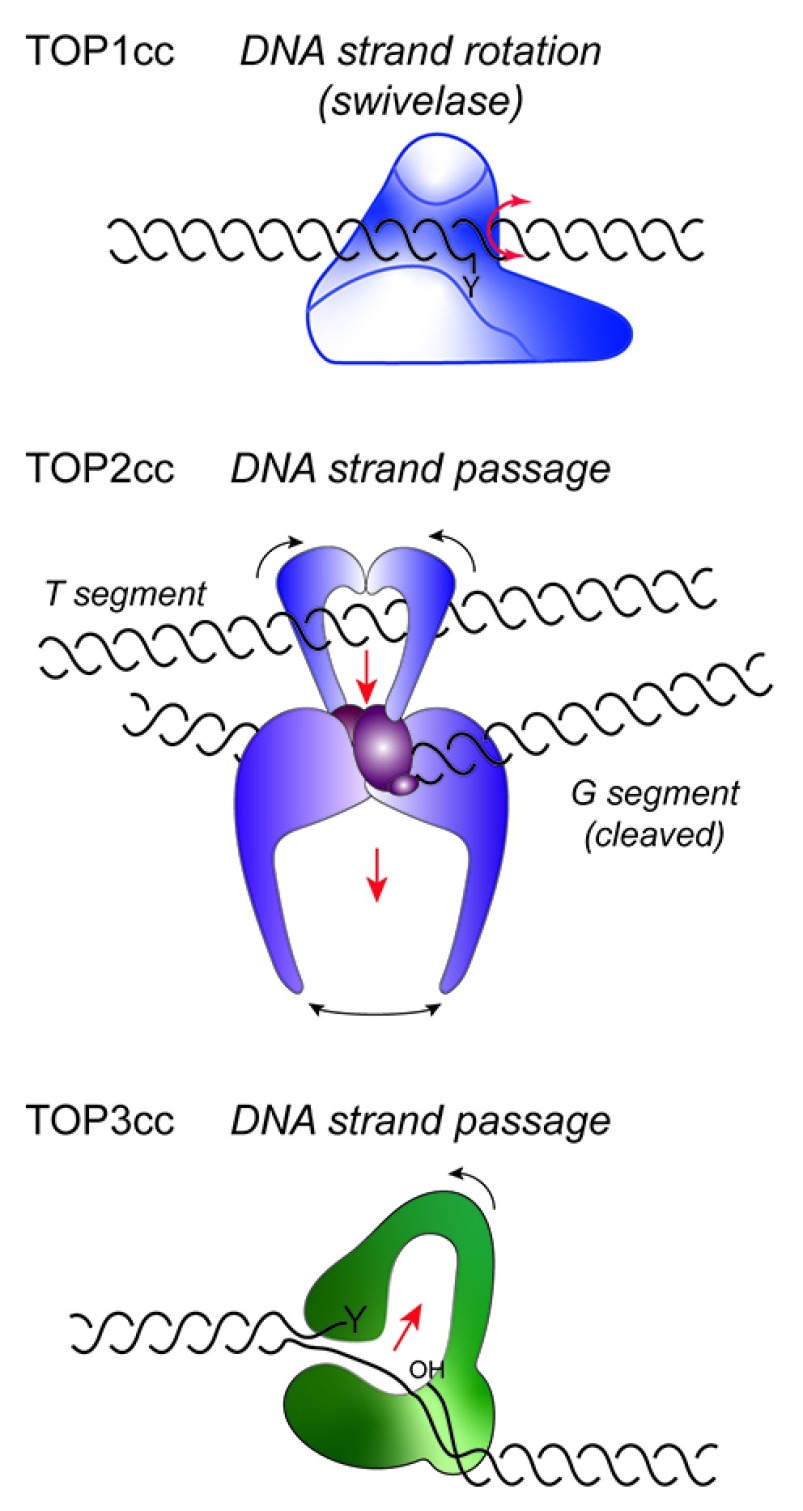
Topoisomerase mechanisms. In the topoisomerase I cleavage complex (TOP1cc) (top), the 3’ DNA end is covalently linked to the active site tyrosine (Y). Changes in the linkage of DNA strands occur through a mechanism of strand rotation, where the untethered 5’ DNA end of the cleaved strand swivels about the noncleaved DNA strand. TOP2 (middle) and TOP3 (bottom) both involve mechanisms of DNA strand transfer. In the case of TOP2cc, the G segment of duplex DNA is cleaved by the two active sites of the homodimer, following capture of the T segment by the closure of the N-terminal ATPase domains. The T segment DNA is then successively passed through the double-strand break in the G segment and out through the bottom dimer interface. For type IA enzymes, depicted for TOP3cc, a single strand of negatively supercoiled DNA is cleaved to form a 5’ phosphotyrosyl bond, while the 3’OH end is held by the enzyme. A conformational change in the protein then allows the intact complementary strand to be passed through the protein-linked break, followed by religation of the cleaved DNA.

As detailed in Table 1, topoisomerases perform critical functions in all kingdoms of life and can be divided into five subfamilies (type IA, IB, IC, IIA, and IIB) based on the number of DNA strands cleaved (one or two, for type I or II, respectively), the nature of the covalent phosphotyrosyl intermediate formed (5’ or 3’ linkage), and other aspects of enzyme structure and catalysis (see Figure 1). Nevertheless, these enzymes all share a common mechanism of transient breakage and rejoining of DNA strand(s).

**Table 1.** DNA topoisomerases.

Subfamily *	Mechanism	Activity ^#^	Representative enzymes	Structure	Organism
Type IA (5’)	Enzyme-bridged single DNA strand passage	Relaxation of (–) DNA	Bacterial DNA topoisomerase I	Monomer	*Escherichia coli*
		Decatenation ^14^	Bacterial DNA topoisomerase III	Monomer	*E. coli*
		Introduce (+)	Archaeal reverse gyrase	Monomer	*Archaeoglobus fulgidus*
		Decatenation, resolve recombination intermediates with helicase ^15^	Eukaryal DNA topoisomerase III	Monomer	*Saccharomyces* *cerevisiae*
			Eukaryal DNA topoisomerase IIIα	Monomer	*Homo sapiens*
		Regulates transcription	Eukaryal DNA topoisomerase IIIβ	Monomer	*H. sapiens*
Type IB (3’)	Enzyme-linked DNA strand rotation	Relaxation of (+) and (–) DNA	Poxvirus DNA topoisomerase I	Monomer	Vaccinia virus
			Trypanosome DNA topoisomerase I	Heterodimer	*Leishmania donovani*
			Eukaryal DNA topoisomerase I	Monomer	*H. sapiens/S. cerevisiae*
			Mitochondrial DNA topoisomerase I	Monomer	*H. sapiens*
Type IC (3’)	Enzyme-linked DNA strand rotation	Relaxation of (+) and (–) DNA	Archaeal DNA topoisomerase V	Monomer	*Methanopyrus kandleri*
Type IIA (5’)	Enzyme-bridged duplex DNA passage	Introduction of (–) into DNA	Bacterial DNA gyrase	A _2_B _2_ heterotetramer	*E. coli*
		Relaxation of (+), decatenation	Bacterial DNA topoisomerase IV	C _2_D _2_ heterotetramer	*E. coli*
		Relaxation of (+) and (–) DNA/decatenation	Eukaryal DNA topoisomerase II	Homodimer	*S. cerevisiae*
			Eukaryal DNA topoisomerase IIα	Homodimer	*H. sapiens*
			Eukaryal DNA topoisomerase IIβ	Homodimer	*H. sapiens*
Type IIB (5’)	Enzyme-bridged duplex DNA passage	Relaxation of (+) and (–)	Archaeal DNA topoisomerase VI	A _2_B _2_ heterotetramer	*Sulfolobus shibatae*
		DNA/decatenation	Plant DNA topoisomerase VI	A _2_B _2_ heterotetramer	*Arabidopsis thaliana*
		Weak relaxation/ decatenation	Bacterial DNA topoisomerase VIII ^16^	Homodimer	*Ammonifex degensii*

*Type I and II enzymes transiently cleave one or two strands of duplex DNA, respectively. As a consequence, type I enzymes catalyze changes in linking number (Lk) in steps of one, while type II enzymes alter Lk in steps of two. Type IA and all type II enzymes form topoisomerase cleavage complexes involving phosphotyrosyl linkages with a 5’ DNA end, while type IB and IC enzymes form 3’ phosphotyrosine bonds. ^**#**^(–) and (+) refer to negatively and positively supercoiled DNA, respectively.

**Figure 2.**
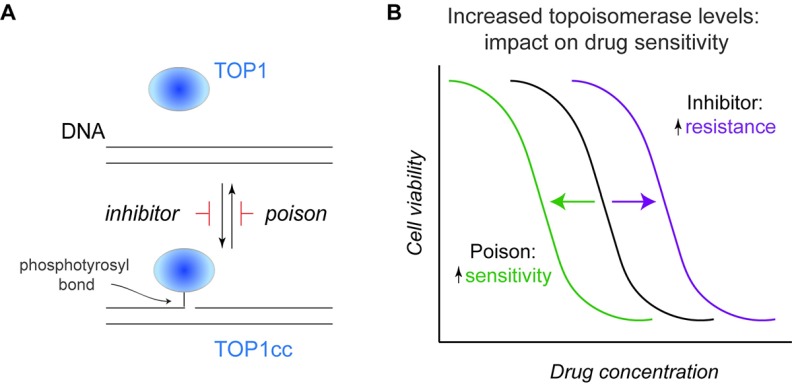
Distinct actions of topoisomerase poisons and inhibitors. ( **A**) As diagrammed for TOP1, a canonical inhibitor would prevent enzyme-mediated cleavage of a single strand of duplex DNA, while a poison (such as camptothecin) acts to stabilize the topoisomerase I cleavage complex (TOP1cc) reaction intermediate, thereby converting a normal enzyme into a source of DNA damage. The same principles apply to TOP2, although, in these instances, the dimeric enzymes produce two enzyme-linked DNA breaks staggered by 4 bp. ( **B**) Based on these distinct modes of action, increased topoisomerase levels in an isogenic cell line would induce opposing effects on drug sensitivity: resistance to an inhibitor versus increased sensitivity to a poison. Shown in this diagram are the dose response curves for killing that result from an increase in topoisomerase levels relative to cells that yield the black curve.

Topoisomerase-linked DNA breaks (topoisomerase-cleavage complexes or TOPccs) are integral to topoisomerase-mediated changes in DNA topology but also pose potential threats to genome integrity. For example, trapping of a TOPcc in advance of the replication machinery or during chromosome segregation, where interwound (or catenated) DNA helices are unlinked by topoisomerases, can have dire effects on genome stability and cell viability. Indeed, topoisomerases are the cellular targets of a wide spectrum of antimicrobial and anticancer agents, which either act to stabilize TOPccs (termed poisons) or otherwise inhibit enzyme catalysis to induce DNA damage ^5– 8^. This difference between poisons and inhibitors is illustrated in Figure 2. In eukaryotes, topoisomerase poisons include camptothecins (topotecan and SN-38, the active metabolite of the drug irinotecan), which stabilize TOP1ccs, and doxorubicin or etoposide, which stabilize TOP2ccs. In addition to these drugs, DNA modifications themselves, such as lesions induced by oxidative damage or ribonucleotides mistakenly incorporated into DNA, may also stabilize TOPccs. Although topoisomerases provide critical solutions to the topological problems imposed by the helical structure of duplex DNA, the hallmark of these activities—the formation of a covalent enzyme–DNA intermediate—constitutes an inherent threat to genome integrity.

In this review, we focus on recent advances in our understanding of topoisomerase function in eukaryotic cells, the therapeutic targeting of topoisomerases in cancer, and the repair pathways that resolve the resulting drug-induced lesions. While highlighting these advances, we also identify unanswered questions that these new findings raise.

## Roles of topoisomerases in nuclear organization and genomic stability

The distinct biological functions of individual topoisomerases and the physiological consequences of altering their activity have been extensively studied (for reviews, see 1– 4, 9). Nevertheless, the technical challenges of assessing local changes in DNA topology in live cells leave perplexing questions regarding topoisomerase function in maintaining chromosome architecture and genome stability. In this section, we summarize recent studies that highlight surprising aspects of eukaryal topoisomerase function.

### Type IB topoisomerases: maintenance of nuclear and mitochondrial genome stability

In eukaryotes, nuclear TOP1 catalyzes the relaxation of local domains of positive and negative supercoils during DNA replication, recombination, transcription, and possibly chromosome condensation ^4, 5, 10^. Stabilization of TOP1ccs by camptothecins during replication is an effective strategy for treating solid tumors and hematologic malignances, as discussed below. During transcription, the phosphorylated C-terminal domain of the catalytic subunit of RNA polymerase II binds and activates TOP1, effectively tethering TOP1 to the transcriptional machinery ^11, 12^. TOP1 then relaxes positive supercoils, which are generated ahead of the transcription complex and could otherwise impede its progress, as well as negative supercoils behind the transcription complex.

In the absence of TOP1, local accumulation of negative supercoils facilitates the formation of R-loops, stable hybrid RNA–DNA duplexes of the nascent RNA transcripts and template strands. R-loops also allow the formation of secondary structures, such as G-quadraplexes and hairpins, in the single-stranded non-template strand. RNase H1 and H2 can degrade RNA in these RNA–DNA heteroduplexes. While genome-wide R-loop mapping indicates context-dependent gains and losses in R-loops when TOP1 is depleted ^13^, it is the increased levels of R-loops and G-quadraplexes that are associated with dysregulation of transcription and replication as well as genome instability.

The misincorporation of ribonucleotides into DNA, at rates approaching 10 ^6^ ribonucleotides per genome per replication cycle ^17^, can also lead to replication stress, single- and double-strand breaks, and small deletions ^18– 20^. Ordinarily these ribonucleotides are removed by the concerted action of RNAse H2, DNA polymerase δ, FLAP endonuclease, and DNA ligase 1 ^17^. However, if ribonucleotides are not removed, TOP1 cleavage of the strand immediately 3’ to the ribonucleotide results in the nucleophilic attack of the 2’OH of the ribonucleotide on the TOP1cc to generate a 2’,3’ cyclic phosphate at the 3’ DNA end and release of TOP1. A second, upstream TOP1 cleavage event can then liberate a short oligo with the modified 3’ end, trapping the TOP1cc across a gap. If DNA strand realignments juxtapose the free 5’OH from the first cleavage and the TOP1cc, enzyme-mediated ligation can produce short deletions. In highly transcribed genes, this TOP1-mediated mutagenesis can be exacerbated by the tethering of TOP1 to RNA polII ^21^.

A recent genome-wide CRISPR screen showed that interruption of genes encoding the three subunits of RNase H2 enhances human cell line sensitivity to the poly(ADP-ribose) polymerase (PARP) inhibitor olaparib ^22^. Further studies attribute this olaparib hypersensitivity to increased ribonucleotide-dependent stabilization of TOP1ccs, which can serve as PARP1 substrates. These observations provide a compelling rationale for inhibiting PARP in order to trigger TOP1cc-initiated killing in cancers with deleted or mutated *RNASEH2B*.

In vertebrates, a second nuclear-encoded type IB topoisomerase (TOP1MT) selectively localizes to mitochondria and catalyzes the relaxation of circular mitochondrial DNA ^23^. Despite its similarity to nuclear TOP1, TOP1MT does not contribute to camptothecin-induced toxicity. Instead, TOP1MT physically associates with mitochondrial ribosome subunits to promote mitochondrial translation, which is critical for hepatocellular carcinoma cell growth ^24^. These findings suggest that inhibition of TOP1MT activity, rather than stabilization of TOP1MTccs, might be an effective strategy for targeting this enzyme to treat some cancers.

### Evolving understanding of eukaryal topoisomerase IIIα and β (TOP3α and β)

Distinct from the swivelase activity ascribed to type IB enzymes, type IA topoisomerases exhibit a mechanism of enzyme-bridged strand passage ( Figure 1) ^25^. As with bacterial TOPA, eukaryotic TOP3α enzymes (including yeast TOP3) preferentially relax highly negatively supercoiled DNA and decatenate duplex DNA molecules tethered by single-stranded DNA interlinks or hemicatenanes ^26^. Differential splicing produces nuclear and mitochondrial isoforms of vertebrate TOP3α. Nuclear TOP3α forms a complex with the BLM helicase and RMI1 and RMI2 proteins ^27^ to resolve double Holliday junctions during recombination ^28^. In contrast, mitochondrial TOP3α decatenates newly replicated mtDNA circles, which are linked by a hemicatenane formed at the origin of replication, to allow segregation of replicated mitochondrial genomes ^29^. Accordingly, TOP3α dysregulation results in human mitochondrial disease.

TOP3β, another type IA topoisomerase encoded by the *TOP3B* gene, binds mRNA and functions during neurodevelopment ^30^. Recent studies, made possible by the development of circular double-stranded and knotted single-stranded RNA substrates, suggest that TOP3β can catalyze RNA topoisomerization ^31– 33^. In multicellular organisms, an association with Tudor domain-containing protein 3 (TDRD3) localizes TOP3β to transcriptionally active chromatin and polyribosomes ^34, 35^. Although type IA enzymes with RNA topoisomerase activity have been detected in all domains of life ^34^, the biological significance of RNA topoisomerization requires further study.

### Contribution of topoisomerase II to chromosome architecture and genomic stability

In eukaryotes, TOP2 is a homodimeric enzyme that relaxes positively or negatively supercoiled DNA and catenates or decatenates duplex DNA via transient breakage of both DNA strands ( Figure 1). Yeast encode a single TOP2, while human cells express TOP2α and TOP2β enzymes, encoded by the *TOP2A* and *TOP2B* genes, respectively. Although human TOP2 enzymes exhibit structural and mechanistic similarities, TOP2α decatenates sister chromatids during chromosome segregation, whereas TOP2β has been implicated in transcription. Several recent studies further define distinct roles of these enzymes in chromosome dynamics.

TOP2β plays a surprising and important role in interphase chromatin organization. High-resolution whole-genome chromatin conformation capture (Hi-C), or *in situ* Hi-C with DNA–DNA proximity ligation, allows chromatin fragments in close proximity to be identified. These techniques have determined that chromosomes are organized into topologically associated domains (TADs) of ~200 kb to 1 Mb, typically bound by chromatin enriched in transcriptionally active genes. According to current models, DNA is actively extruded through one or paired cohesin rings to generate TADs until DNA bound by the CCCTC binding factor (CTCF) is encountered ^36, 37^. Recent studies suggest that CTCF becomes associated with loop anchors and unidirectionally halts DNA extrusion. TOP2β is then recruited to loop anchors to alleviate the positive supercoils induced by cohesin-derived DNA extrusion. The resulting TOP2β-induced breaks are transcription independent but correlate with cohesin ^38^. At a low frequency, unresolved TOP2βccs at these loop anchors can also lead to DNA breakage and translocations ^39^. Thus, TOP2β involvement in topological dynamics associated with chromosome organization contributes somewhat unexpectedly to chromosome breakage and rearrangements.

During chromosome segregation, intertwined DNA duplexes (catenanes) are resolved or decatenated by TOP2 in yeast and TOP2α in human cells. TOP2 enzymes can also readily catenate DNA duplexes in close proximity. Yet increased positive supercoiling drives decatenation, based in part on an intrinsic enzyme bias towards decatenation. A persistent question, then, has been the source of this positive supercoiling to drive decatenation. In yeast, condensin-mediated positive DNA supercoiling increases as cells enter mitosis ^40^. In human cells, we now know that this positive supercoiling reflects the action of TOP3α, which (as part of the TRR complex with RMI1 and RMI2) associates with the Plk1-interacting checkpoint helicase (PICH) to produce extremely high-density positive supercoils ^41^. Subsequent relaxation of negative supercoils by TOP3α results in the accumulation of positive supercoils, which drives decatenation by TOP2α. These studies provide the first evidence for topoisomerase-induced stable domains of positive supercoils in eukaryotic cells and illustrate how DNA extrusion can be locally harnessed to drive chromosome disjunction.

## Recognition and resolution of TOPccs

During their catalytic cycles, all topoisomerases transiently form covalent linkages between active site tyrosines and DNA ^42– 45^. While the vast majority of these TOPccs are normally resolved by completion of the catalytic cycle, there is increasing interest in the question of what happens when the TOP1 or TOP2 catalytic cycle is slowed or impaired. These issues are particularly critical in the context of anticancer drugs ( Table 2) and endogenous DNA lesions (abasic sites, oxidized nucleotides, and alkylated bases), which stabilize or trap TOPccs ^46– 50^. Thus, the way in which cells deal with TOPccs has biological and pharmacological implications.

**Table 2.** FDA-approved anticancer drugs that increase TOP1- or TOP2-containing DPCs.

Drug	Target	Clinical status	Clinical uses	Refs
Irinotecan	TOP1	FDA approved	Colorectal, pancreatic, and lung cancers	61
Topotecan	TOP1	FDA approved	Ovarian, cervical, and small cell lung cancer	62, 63
MM398	TOP1	FDA approved	Pancreatic cancer with 5FU and leucovorin	64, 65
Etoposide	TOP2	FDA approved	Acute leukemia, lymphoma, testicular cancer, and lung cancers	66– 69
Doxorubicin	TOP2	FDA approved	Breast and bladder cancers, leukemias, lymphomas, and neuroblastoma	66, 69, 70
Daunorubicin, idarubicin	TOP2	FDA approved	Acute leukemia	66, 69, 70
Mitoxantrone	TOP2	FDA approved	Acute leukemia	66, 69, 71, 72

5FU, 5-fluorouracil; DPC, DNA–protein crosslinks; FDA, US Food and Drug Administration; TOP1, topoisomerase I; TOP2, topoisomerase II

### TOP1cc removal: multiple pathways and unanswered questions

DNA–protein crosslinks (DPCs) include not only TOP1ccs, but also crosslinks induced by aldehyde products of demethylation reactions, cisplatin, UV light or ionizing radiation, and trapping of DNA methyltransferases covalently bound to 5-aza-cytosine (reviewed in 51– 53). Distinct repair pathways have evolved to resolve these DPCs; however, TOPccs present unique challenges because they also involve protein-linked DNA breaks. Recent studies have provided new insight into the action of tyrosyl-DNA phosphodiesterases 1 and 2 (TDP1 and TDP2, respectively) and DNA-dependent proteases such as SPARTAN (also known as SPRTN) that recognize and reverse persistent TOP1ccs.

Several lines of evidence implicate TDP1 in TOP1cc removal.TDP1 can de-esterify peptidic tyrosine-phosphoesters ^54, 55^, and TDP1 knockdown results in increased foci containing the TOP1 active site peptide covalently bound to DNA ^56^. Earlier studies suggested that TPD1 efficiently removes short TOP1 peptides from DNA but is less efficient at removing longer peptides or full-length TOP1 ^57^. However, recent studies of TDP1 mutants suggest that full-length TOP1 can, in fact, be released from chromatin-bound TOP1ccs in yeast and human cells ^58, 59^.

The observation that TDP1 knockdown or knockout has little impact on yeast or mammalian cell sensitivity to camptothecins ^55, 60^ suggested early on that there must be redundant or overlapping repair pathways. In the absence of TDP1, the 5’-tyrosyl phosphodiesterase TDP2 ^73, 74^ and a pathway involving the repair proteins XPF and ERCC1 ^75^ participate in TOP1cc removal. An additional pathway involves cleavage of the adducted DNA by the nuclease MUS81 followed by polymerization and ligation across the resulting gap ^76^.

Conditions that promote the use of one pathway over another are still being elucidated. Poly(ADP-ribosyl)ation of TDP1 appears to influence this choice ^75^. In addition, the deubiquitylase UCHL3 was recently shown to regulate TDP1 proteostasis ^77^, implicating ubiquitin-dependent regulation of TDP1 in the repair of camptothecin-induced TOP1ccs.

Emerging results also suggest a role for proteases in the removal of TOP1ccs. Although early studies implicated the proteasome in this process ^78– 81^, the observation that proteasome-mediated TOP1 degradation occurs only at micromolar camptothecin concentrations and not at more clinically relevant low nanomolar concentrations ^56^ calls this model into question. Instead, the nuclear metalloproteinase SPARTAN, which contains a ubiquitin-binding domain and a single-stranded DNA-binding motif ^82^, has recently been shown to reverse TOP1ccs trapped by normal DNA metabolism or nanomolar camptothecin concentrations ^56, 82– 84^. In *Saccharomyces cerevisiae*, the SPARTAN homolog Wss1 is critical for survival after camptothecin treatment, and the recombinant protease is able to cleave TOP1ccs ^85^. Likewise, *Sprtn* downregulation increases TOP1ccs in murine fibroblasts ^56^ and enhances camptothecin sensitivity *in vitro* ^56, 82, 83^. Mice bearing a hypomorphic *Sprtn* allele contain increased hepatocyte TOP1ccs and develop hepatic neoplasms ^56^, which recapitulates Ruijs-Aalfs syndrome, a disorder characterized by germline *SPRTN* mutations, genomic instability, and early onset hepatocellular carcinoma ^86– 88^. This hepatocyte-specific pathology is, at present, poorly understood. Higher TOP1 protein levels ^56^ might contribute to preferential trapping of TOP1ccs in Spartan-deficient hepatocytes, but the possibility that alternative proteases facilitate the removal of TOP1ccs in other tissues also merits investigation. Additional unresolved issues include i) the coupling between proteases and phosphodiesterases or nucleases and ii) the relative contributions of protease-dependent versus protease-independent pathways in TOP1cc removal.

### Recognition of trapped TOP1ccs: a plethora of modifications

A particularly perplexing question is how do trapped TOP1ccs come to be marked for repair or proteolytic degradation? Post-translational modifications of TOP1 and TOP1ccs by ubiquitin ^78– 81^, ubiquitin-like modifiers ^89, 90^, and phosphorylation ^91– 93^ have been reported, but the physiological relevance of these modifications to TOP1cc resolution is complicated by the use of high camptothecin concentrations.

In this context, studies implicating the small ubiquitin-like modifier (SUMO) in TOP1 action might be pertinent. TOP1 is modified by SUMOylation in CPT-treated yeast and mammalian cells ^89, 90, 94^. In addition, downregulation or mutation of the sole SUMO E2 ligase Ubc9 is associated with TOP1cc stabilization and enhanced camptothecin toxicity ^89, 90, 95, 96^. However, recent studies ascribe these effects to a change in Ubc9 substrate specificity ^97^, consistent with more global changes in SUMOylation of other proteins involved in the DNA damage response and not a direct effect on TOP1.

It is also worth noting that TOP1cc degradation by Wss1 (yeast SPARTAN) occurs in a SUMO-dependent fashion ^98^, while SPARTAN preferentially binds ubiquitin through a UBZ domain, and its activity is regulated by deubiquitinylation ^82, 99^. These differences in the SUMO- versus ubiquitin-mediated regulation of Wss1 and SPARTAN, and the inability of SPARTAN to complement *wss1*Δ yeast cells, led Mailand and colleagues to examine SUMO-dependent responses to various DPCs ^100^. Their studies implicate SprT metalloproteases of the ACRC/GCNA-1 family in SUMO-dependent resolution of DPCs. While it remains to be determined if GCNA-1 family proteases impact sensitivity to drug-stabilized TOP1ccs, these observations support the notion that other, as-yet-uncharacterized metalloproteases may regulate cellular responses to topoisomerase-mediated DNA damage via distinct ubiquitin-like protein modifications. The potential therapeutic implications of these recently recognized repair pathways remain to be more fully investigated.

### Extending the paradigm to TOP2cc

The machinery responsible for removing trapped TOP2ccs is even less clearly defined. Proteasomal degradation of TOP2 after teniposide treatment has been reported ^101^, contributing to a model in which collisions between advancing transcription complexes and TOP2ccs result in irreversibly trapped TOP2–DNA complexes, which are marked by ubiquitylation and degraded by the proteasome.

More recent studies have identified several alternatives to this model. First, SPARTAN knockdown results in slightly increased levels of TOP2ccs and etoposide sensitivity ^83^, suggesting SPARTAN might degrade TOP2 before removal of the active site peptide from DNA. Contrary to this model, however, increased TOP2ccs were not observed in MEFs conditionally deleted for *Spartan*, and MEFs harboring a hypomorphic *Spartan* allele were not hypersensitive to etoposide ^56^. Thus, the role of SPARTAN and other nuclear metalloproteinases in the removal of trapped TOP2cc requires further clarification.

Trapped TOP2ccs may also be removed without TOP2 proteolysis. The MRE11 nuclease has been implicated in the removal of TOP2 from DPCs ^102, 103^. Moreover, TDP1 ^104, 105^ and TDP2 ^73, 106^ have both been reported to release the TOP2 active site peptide when it is linked to 5’OH of the DNA backbone. In particular, TDP2 can reverse covalent binding of TOP2α or TOP2β to a suicide DNA substrate, and this activity increases up to 1000-fold in the presence of the SUMO E3 ligase ZNF451 owing to increased binding of TDP2 to SUMOylated TOP2 ^107^.

Two recent studies further suggest that coordination of SUMO- and Ub-dependent TOP2 modifications may be critical for genomic stability. In etoposide-treated fission yeast, the DNA translocase Rrp2 binds to SUMOylated TOP2ccs and prevents recruitment of the SUMO-dependent E3 ubiquitin ligase STUbL, thereby preventing STUbL-mediated TOP2 ubiquitinylation and degradation ^108^. Instead, Rrp2 facilitates the eviction of intact TOP2 from the DNA and concomitant DNA resealing, thereby increasing genomic stability and etoposide resistance. In other studies, the Smurf2 E3 ubiquitin ligase was shown to switch the pattern of TOP2α modification from K48 polyubiquitylation that promotes proteasomal degradation to monoubiquitylation, which leads to increased TOP2α protein levels, suppression of anaphase bridge formation, and etoposide resistance ^109^.

In summary, although multiple pathways have been implicated in the reversal of trapped TOP2ccs, other studies suggest that protecting TOP2ccs from proteolytic degradation is also critical for maintaining genome stability. Further studies are required to assess whether distinct pathways are called into play in response to different levels of DNA damage, as appears to be the case with TOP1, or whether current inconsistencies reflect differential expression of pathway components in different cell types.

## Translating biological knowledge into improved therapy

The TOP1- and TOP2-targeted drugs in Table 2 all have activity in the clinical setting, albeit with narrow therapeutic windows. Accordingly, recent efforts to develop topoisomerase poisons ^66^ into more effective antineoplastic agents have tried to address a series of issues.

### Can the delivery and efficacy of topoisomerase poisons be improved?

Consistent with observations that TOP1 and TOP2 poisons are preferentially toxic during S phase, classic studies demonstrated that the administration of irinotecan on a five-times-daily schedule for 2 weeks is more active against human cancer xenografts than less-protracted schedules ^110^. Etoposide administered every other day for three doses is likewise more effective against L1210 murine leukemia than a higher dose administered once. In the clinical setting, these observations have been translated into protracted schedules of both irinotecan ^111^ and etoposide ^112^. Because these prolonged schedules can be inconvenient and toxic ^111^, there has been an ongoing search for alternatives, including new topoisomerase poisons, drug formulations that extend the half-life of TOPccs, and strategies to increase tumor-selective drug delivery ( Table 3) ^113^.

**Table 3.** Emerging inhibitors of mammalian TOP1 or TOP2 ^a^.

**New TOP1 inhibitors**	Compound	Unique features and references
	STA-8666	1. Covalent fusion of STA-8663 (HSP90 inhibitor) and SN-38 through a cleavable chemical linker 2. Prolonged tumor exposure relative to irinotecan *in vivo* 3. Very active against small cell lung cancer and sarcoma xenografts ^127, 128^
	Indenoisoquinolines	1. Stabilize TOP1–DNA covalent complexes but lack the lactone ring of camptothecin and its derivatives 2. As a consequence, the TOP1–DNA covalent complexes do not peak and then decrease as they do with camptothecins ^115, 129^
	7-aza-indenoisoquinolines	1. These non-camptothecin agents lack the lactone ring that is part of the camptothecin backbone ^130^
	Fluoroindenoisoquinolines	1. These indenoisoquinoline derivatives contain fluorine in place of methoxy side chains and are more potent than the parent compounds ^131, 132^
**Novel formulations of** **TOP1 inhibitors**	**Antibody–drug** **conjugates**	
	DS-8201a	1. Deruxtecan (CPT derivative) covalently coupled to anti-HER2 antibody through cleavable linkage 2. Targets HER2-expressing tumors with activity against low-expressing tumor cells ^123, 124^ 3. Enhances antitumor immunity in mouse model ^133^ 4. Evidence of activity in clinical trials in HER2 ^+^ trastuzumab emtansine-resistant breast cancer ^125^ and gastric cancer ^126^
	U3-1402	1. Deruxtecan covalently coupled to anti-HER3 antibody through cleavable linkage 2. Targets HER3-expressing tumor cells ^134^
	Sacituzumab Govitecan	1. SN-38 covalently coupled to antibody to human trophoblast cell surface antigen (TROP2), a glycoprotein found on several solid tumors ^135– 137^ 2. Objective response rates in phase II clinical trials in triple negative breast cancer (30%) ^138, 139^, non-small cell lung cancer (19%) ^140^, and metastatic small cell lung cancer (14%) ^141^ 3. Synergizes with PARP inhibitors in triple-negative breast cancer independent of *BRCA1/2* mutation status ^142^
	**Novel formulations**	
	Di-SN38-phosphatidylcholine	1. Liposomes of two SN-38 molecules covalently bound to phosphatidylcholine 2. Extended half-life in mice ^143^
	Camptothecin or SN-38 in functionalized carbon nanotubes	1. Encapsulation in carbon nanotubes bearing carboxylate groups on their surfaces enhances solubility in aqueous solution while maintaining antiproliferative effects *in vitro* ^144^
	Camptothecin in β- cyclodextrin nanosponges	1. Increased solubility and protection from degradation compared to parent CPT 2. Active against prostate cancer and anaplastic thyroid carcinoma *in vitro* ^145, 146^
**Novel TOP2 inhibitors**	**Compound**	
	F14512	1. Polyamine-conjugated etoposide derivative 2. Depends on TOP2A for killing 3. TOP2cc last longer and do not depend on TDP2 for removal ^147^ 4. Marrow suppression was dose limiting in a phase I study ^148^
	Pixantrone	1. Selectively targets TOP2A 2. Diminished oxidative stress relative to anthracyclines because it binds less Fe(II) ^149^
	Vosaroxin	1. Anti-cancer quinolone derivative 2. DNA intercalator, with a possible role in sequence-specific TOP2 poisoning ^150, 151^
	**Novel formulations**	
	Dimethylepipodophyllotoxin coupled to specific nucleotide sequence	1. Demonstrate somewhat selective cleavage of complementary sequence, raising possibility of using coupled oligonucleotides to target TOP1 or TOP2 poisons to specific sequences ^152^

^a^ https://clinicaltrials.gov TOP1, topoisomerase I; TOP2, topoisomerase II; TOP2cc, topoisomerase II cleavage complex

Among the new classes of TOP1 or TOP2 poisons, TOP1-directed indenoisoquinolines ^114, 115^ are furthest along in development. These agents, which lack a lactone ring and, in contrast to camptothecin derivatives, do not exist in equilibrium between an active agent and inactive derivative ^115^, exhibit promising activity against canine lymphomas ^116^. Assessments of their activity in humans are awaited with interest.

An alternative approach involves new formulations that extend tumor exposure. MM398, a nanoliposomal irinotecan formulation ^117^, gained FDA approval in combination with 5-fluorouracil and leucovorin for gemcitabine-resistant pancreatic cancer ^64, 65^. In contrast, NKTR-102, a PEGylated irinotecan, exhibited disappointing activity in breast and ovarian cancer ^118, 119^. Whether the different outcomes for these two sustained-release irinotecan formulations reflect differences in pharmacokinetics, intratumoral accumulation, or simply choice of tumors studied is not clear.

Santi and coworkers developed an ultra-long-acting Prolynx PEG~SN-38 that accumulates in tumors and delivers active SN-38 rather than the prodrug irinotecan ^120^. Liposomal topotecan formulations are also being developed ^121^. Whether the promising preclinical activity seen in experimental tumors, which is thought to reflect enhanced permeability and retention of nanoformulations ^122^, can be translated into increased clinical efficacy remains to be determined.

Antibody–drug conjugates ( Table 3) also hold the promise of more selectively delivering TOP1 poisons to tumor cells. DS-8201 ^123, 124^, a conjugate of the TOP1 poison deruxtecan with the anti-HER2 antibody trastuzumab, is currently undergoing extensive preclinical and early clinical testing ( www.ClinicalTrials.gov). Promising clinical activity has been observed in trastuzumab-resistant breast and gastric cancers ^125, 126^. An immunoconjugate of SN-38 and antibody to human trophoblast cell surface antigen 2 (TROP2), a glycoprotein found on several solid tumors ^135– 137^, likewise exhibits promising activity in breast ^138, 139^ and lung cancers ^140, 141^ ( Table 3).

### Should topoisomerase poisons and DNA damage response modulators be combined?

Because TOP1 and TOP2 poisons lead to DNA damage, there has been substantial interest over the past few years in combining these drugs with several different DNA damage response modulators.

***PARP inhibitors.*** PARP inhibitors (PARPis), which inhibit PARP1 as well as other PARP family members ^153^, are FDA approved for high-grade serous ovarian cancer, germline *BRCA1/2*-mutated breast cancer, and *BRCA1/2*-mutated castration-resistant prostate cancer ^154– 158^. Additional studies identified a role for PARP1 in stabilizing ^159– 161^ and restarting ^162, 163^ stalled replication forks, including forks stalled by TOP1ccs. Consistent with these studies, Curtin *et al*. demonstrated that PARPis increase killing by TOP1 but not TOP2 poisons ^164^. This TOP1 poison/PARPi synergy likely results from trapping of inhibited PARP ^165, 166^ at sites of TOP1ccs or TOP1cc-induced DNA damage ^167^, perhaps in concert with diminished recruitment of TDP1 to TOP1ccs ^75^.

Building on xenograft studies ^168, 169^, several clinical trials have evaluated TOP1 poison/PARPi combinations ( Table 4). Most started with myelosuppressive topotecan or irinotecan regimens ^170^. Because PARPis also suppress bone marrow function ^171^, it is not surprising that profound myelosuppression occurs with these combinations, limiting drug doses that can be safely administered together ( Table 4). In contrast, by starting with a less myelosuppressive weekly topotecan regimen ^172^ and only administering PARPi for 72 hours around each topotecan dose to maximize the synergy, Wahner Hendrickson and coworkers were able to escalate topotecan and veliparib to three-quarters of the single-agent MTDs ^173^. Whether the approach of i) using a less myelosuppressive TOP1-directed regimen and/or ii) giving intermittent PARPi timed to coincide with maximal TOP1cc stabilization will be an effective way forward with TOP1 poison/PARPi combinations remains to be further assessed.

**Table 4.** Recently described combinations of TOP1 or TOP2 poisons with other agents.

	Topoisomerase poison	Other agent(s)	Observations		Ref
**Preclinical** **studies**		**PARP inhibitors**			
	Topotecan	Veliparib	Synergy observed at concentrations far below those required to inhibit most PARP activity Transfection with catalytically dead PARP1 also sensitizes		167
	Camptothecin	Niraparib			
		**ATR inhibitor**			
	Topotecan	Berzosertib	Sensitization to TOP1 inhibitor in multiple ovarian cancer cell lines		182
	Irinotecan	Berzosertib	Enhanced antitumor effects in colorectal xenografts		183
		**CHK1 inhibitors**			
	SN-38	MK-8776	Maximum sensitization when CHK1 inhibitor administered 24 hours after TOP1 poison *in vitro*		181
	Irinotecan	AZD7762	Sensitization observed in triple-negative breast cancer xenografts		180
		**CDK inhibitors**			
	Irinotecan	Palbociclib	Sensitization of colon cancer cells *in vitro* regardless of presence of hypoxia		218
		**Immune checkpoint inhibitor**			
	DS-8201a	Anti-PD-L1	DS-8201a enhances dendritic cell function		133
	Irinotecan	Anti-PD-L1	Irinotecan suppresses regulatory T cells and upregulates MHC class I		198
**Clinical studies**		**PARP inhibitors**	**Clinical trial observations**	**Phase**	
	Topotecan five times daily	Veliparib	Dose-limiting hematological AEs Five dose de-escalations to find tolerable dose	I	219
	Topotecan three times daily	Olaparib	Dose-limiting hematological AEs	I	220
	Topotecan weekly	Veliparib	Dose-limiting hematological AEs	I	173
	Irinotecan, day 1 and day 8 every 21 days	Veliparib	Dose-limiting GI and hematological AEs	I	221
	Irinotecan every 2 weeks	Olaparib	Dose-limiting GI and hematological AEs	I	222
		**CHK1 inhibitor**		
	Irinotecan	AZD7762	Dose-limiting cardiotoxicity	I	184
		**ATR inhibitor**			
	Topotecan	Berzosertib	Dose-limiting hematological AEs	I	188

AEs, adverse effects; GI, gastrointestinal; MHC, major histocompatibility complex; PARP, poly(ADP-ribose) polymerase; TOP1, topoisomerase I; TOP2, topoisomerase II

***Combinations with ATR and CHK1 inhibitors.*** Stalled replication forks activate the replication checkpoint, a biochemical pathway involving the DNA damage-activated kinases ATR and CHK1 that inhibits new origin firing, stabilizes stalled forks, and increases DNA repair ^174– 176^. Consistent with a role for this pathway in cellular recovery from TOP1cc-induced damage ^177, 178^, inhibition of CHK1 ^179– 181^ or ATR ^182, 183^ sensitizes cancer cells to TOP1 poisons *in vitro* and in xenografts. Earlier development of a CHK1 inhibitor/TOP1 poison combination ^184^ was abandoned because of off-target cardiac toxicities of the CHK1 inhibitor ^185^. More recent studies have examined ATR inhibitors (e.g. M6620 and AZD6738) ^186, 187^ with TOP1 poisons. Reportedly, an M6620/topotecan combination was well tolerated, except for myelosuppression, and induced partial responses in two out of 21 (9.5%) patients ^188^. A phase II trial of this combination in small cell lung cancer (ClinicalTrials.gov identifier: NCT02487095) and a phase I trial of an irinotecan/M6620 combination (NCT02595931) are ongoing.

***TOP1 poison/immune checkpoint inhibitor combinations.*** While immune checkpoint blockade is highly active in certain solid tumors ^189, 190^, many common cancers respond poorly. However, recent studies suggest that DNA damage can stimulate immune responses through multiple mechanisms. First, release of DNA to the cytosol after DNA damage ^191^ activates the stimulator of interferon genes (STING) pathway ^192, 193^, leading to the production of pro-inflammatory cytokines. Second, DNA damage-induced release of tumor cell microvesicles can increase immune activation ^194, 195^. Third, DNA damage increases antigen presentation on tumor cell MHC class I molecules, leading to enhanced dendritic cell activation and T cell responses ^196^. Importantly, these changes have been observed after treatment with TOP1 poisons, potentially contributing to the synergy observed when irinotecan or DS-8201a is combined with anti-PD-1 *in vivo* ^133, 197, 198^. Clinical trials are also evaluating TOP2 poisons in combination with PD-1 antibodies ( www.ClinicalTrials.gov).

### Predicting response to topoisomerase poisons

Given the toxicities of topoisomerase poisons, the ability to predict responses and avoid treatment of patients unlikely to benefit would represent a major advance. In isogenic yeast ^199, 200^ or mammalian cells ^201, 202^, elevated TOP1 or TOP2 levels are associated with increased killing by topoisomerase poisons ( Figure 2). Additional studies indicate that high TOP1 expression correlates with improved colorectal cancer response to irinotecan ^203– 206^ and *TOP2* gene amplification is associated with improved breast cancer response to doxorubicin ^207, 208^. However, expression and response are not so tightly correlated that outcomes of individual patients can be predicted from expression data alone.

The frequent occurrence of transport-mediated resistance raises the possibility that responses might be better predicted by assaying TOPccs after the first dose of therapy. While earlier techniques for measuring TOPccs were labor intensive and nonspecific, a recently described antibody to TOP1ccs ^209^ opens the possibility of specific, quantitative assays to address the relationship between TOP1ccs and response to TOP1 poisons. Unfortunately, similar reagents to assess TOP2ccs are not currently available.

It is possible that factors other than TOPccs will need to be assessed to predict drug responses. Homologous recombination (HR) defects convey heightened sensitivity to TOP1 and TOP2 poisons in yeast ^210^ and mammalian cells ^167, 211– 213^. Moreover, *BRCA1*- or *BRCA2*-mutant ovarian cancers have a higher response rate to liposomal doxorubicin ^214, 215^. Likewise, breast cancers deficient in BRCA1 or HR activity respond better to anthracycline-based neoadjuvant therapy ^216, 217^. In contrast, *BRCA1/2* mutation status was not correlated with response to the TOP1 poison topotecan administered alone ^214^ or in combination with PARPi ^173^. Thus, HR status might need to be considered in predictive algorithms, but the impact of HR status might also vary by drug class.

## Challenges for the coming decade

As indicated above, recent advances bring into focus a number of topics for future investigation. First, the cellular functions of topoisomerases are incompletely understood, in part because DNA topology still cannot be visualized in intact cells. Second, based on provocative examples, cooperation between various topoisomerases and other enzymes requires further study. Third, when TOPccs are trapped, we still have only rudimentary understanding of the processes that reverse these DPCs and limited insight into the factors that dictate choice between overlapping repair pathways. Finally, even though topoisomerase-directed drugs exhibit anti-neoplastic properties, patients would benefit from more efficacious schedules, more selective delivery of active agents to tumor cells, and potentially bioassays that accurately predict responses to topoisomerase-directed therapy.
